# Axonal branching in lateral olfactory tract is promoted by Nogo signaling

**DOI:** 10.1038/srep39586

**Published:** 2016-12-21

**Authors:** Masumi Iketani, Takaakira Yokoyama, Yuji Kurihara, Stephen M. Strittmatter, Yoshio Goshima, Nobutaka Kawahara, Kohtaro Takei

**Affiliations:** 1 Molecular Medical Bioscience Laboratory, Department of Medical Life Science, Yokohama City University Graduate School of Medical Life Science, Yokohama 230-0045, Japan; 2 Department of Neurosurgery, Yokohama City University Graduate School of Medicine, Yokohama 236-0004, Japan; 3 Department of Neurology and Section of Neurobiology, Yale University School of Medicine, New Haven, CT 06520, USA; 4 Department of Molecular Pharmacology and Neurobiology, Yokohama City University Graduate School of Medicine, Yokohama 236-0004, Japan

## Abstract

Mitral cells are major projection neurons of the olfactory bulb (OB) that form an axonal bundle known as the lateral olfactory tract (LOT). After axonal bundle formation, collateral branches sprout from primary axons of the LOT. Recently, we identified LOT usher substance (LOTUS) as an endogenous Nogo receptor-1 (NgR1) antagonist and demonstrated that LOTUS contributes to the formation of the LOT axonal bundle. Immunoblots revealed that the expression level of Nogo-A in the OB developmentally increased during axonal collateral formation. Next, we found that the axonal collateral branches were increased in cultured OB neurons from LOTUS-knockout (KO) mice, whereas they were decreased in cultured OB neurons from NgR1-KO mice. Knockdown of Nogo-A in cultured OB neurons reduced the number of axonal collateral branches, suggesting that endogenous Nogo-A induces axonal branching. Finally, the collateral branches of the LOT were increased in LOTUS-KO mice, whereas those in NgR1-KO mice were decreased. Moreover, the abnormal increase of axonal branching observed in LOTUS-KO mice was rescued in the double mutant of LOTUS- and NgR1-KO mice. These findings suggest that Nogo-A and NgR1 interactions may contribute to axonal branching in LOT development.

The olfactory bulb (OB) is the first relay for olfactory information. It receives sensory inputs from olfactory receptor neurons and processes this information before sending it to the olfactory cortex. During development, axons from the olfactory receptor neurons exit the olfactory epithelium and grow towards the OB anlage^1^, where they synapse on the dendrites of mitral and tufted cells. These cells project axons into a very narrow part of the telencephalon, and their axons form a fasciculated axonal bundle known as the lateral olfactory tract (LOT). The developing LOT grows away from the midline, extends laterally and elongates caudally at the surface of the telencephalon^2^,^3^. The axons of early-generated mitral and tufted cells emerge from the OB at embryonic day (E) 12 in the mouse, and the main shaft of the LOT is formed during the following 2 days. After that, collateral branches sprout from the primary axons of the LOT^4^,^5^. These collateral branches invade in a precise rostro-caudal order, successively entering the anterior olfactory nucleus, piriform and entorhinal cortex, olfactory tubercle, and anterior cortical as well as posterolateral nuclei of the amygdala^3^. These collateral branches are the only connections of the mitral and tufted cell axons with the olfactory cortex^3^,^4^. Recent studies have revealed some of the molecular mechanisms regulating LOT axon guidance and collateral branching^5^,^6^,^7^,^8^. Previously, we have reported that LOT usher substance (LOTUS) contributes to LOT axonal bundling through its antagonism to the Nogo receptor-1 (NgR1)^9^. LOTUS is a membrane-bound and/or secreted protein, and NgR1 is a glycosylphosphatidylinositol-anchored protein^10^, and they are coordinately expressed on axons of the OB neurons^9^. NgR1 is a common receptor for axonal outgrowth inhibitors such as Nogo^10^, myelin-associated glycoprotein (MAG)^11^, oligodendrocyte myelin glycoprotein (OMgp)^12^, B-lymphocyte stimulator (BLyS)^13^ and chondroitin sulfate proteoglycans (CSPG)^14^.

We recently discovered that LOTUS not only inhibited the signaling of Nogo but also MAG, OMgp and BLyS through blocking the interactions between these ligands and NgR1^15^. However, the physiological roles of Nogo and NgR1 are not well known in the developing brain. Recent studies have shown that Nogo-A induces axonal branching in cultured dorsal root ganglion neurons^16^ and cultured midbrain neurons^17^. In contrast, exogenous Nogo-A inhibits axonal branching in cultured hippocampal neurons^18^. Thus, Nogo-NgR1 signaling plays a key role in axonal branching of the developing brain. As mentioned above, we previously found that the LOTUS-NgR1 interaction induced axonal bundling of LOT through antagonism of NgR1 function^9^. However, the molecular mechanisms of LOT formation, including axonal branching, remain largely unknown. Here, we have shown that LOTUS, NgR1 and Nogo-A are expressed in the OB and LOT during the period of axonal branching (E16–18) and examined how axonal branching is formed even late in development.

## Results

### The expression level of Nogo-A increases in the mouse OB during axonal collateral formation of the LOT

**Figure 1:**
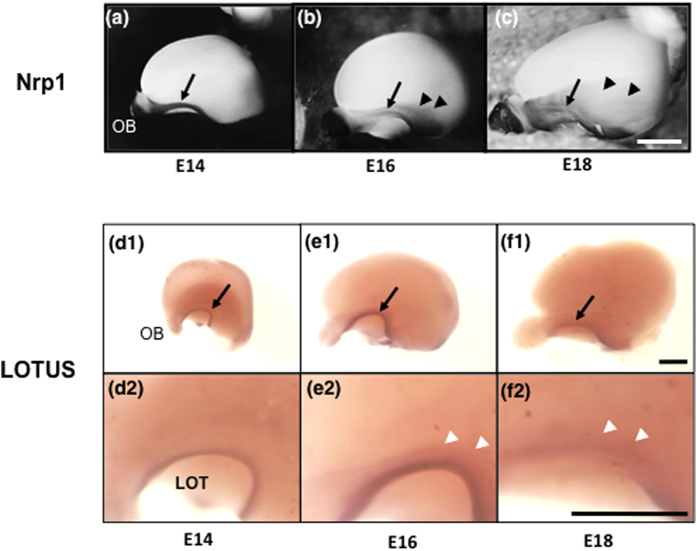
Expression of LOTUS in the developing LOT. (**a–f**) Lateral views of the developing mouse forebrain. (**a–c**) Immunohistochemistry of Nrp1, a LOT marker. Collateral branches (arrowheads) of the LOT (arrow) are observed from E16. (**d–f**) Distribution of LOTUS in the developing mouse forebrain. (**d2–f2**) are higher magnification images of (**d1–f1**). Scale bar: 1 mm, arrows: LOT, arrowheads: collateral branches of the LOT.

A previous study reported that collateral branches of the LOT emerge at E16^4^. First, we confirmed that collateral branches sprout from the primary axons of the LOT, as visualized by immunohistochemistry with the LOT marker molecule Neuropilin-1 (Nrp1) in whole-mount samples of mouse brains (Fig. 1). Collateral branches were not observed at E14 (Fig. 1a) but began to sprout from the primary axons of the LOT at E16 (Fig. 1b) and were elongated at E18 (Fig. 1c). We also examined the expression and distribution of LOTUS in the LOT from E14 to E18 with immunohistochemistry and found that LOTUS was distributed in the axonal bundle of the LOT from E14 to E18 and the axonal collaterals of the LOT after E16 (Fig. 1d–f).

**Figure 2:**
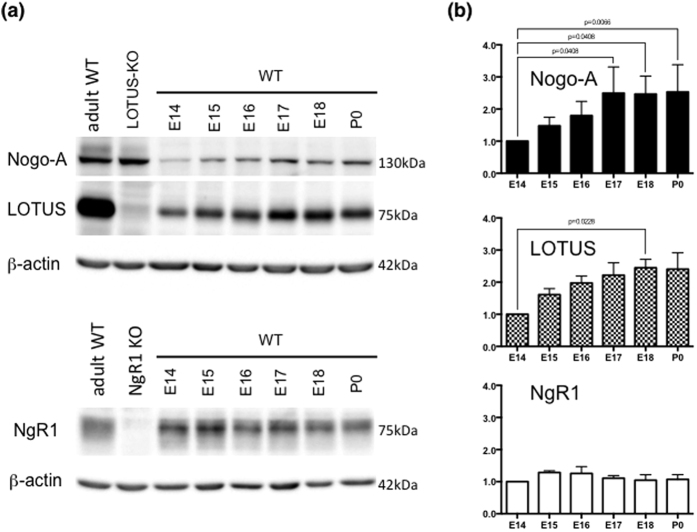
Expression of Nogo-A, NgR1 and LOTUS in the OB. (**a**) Immunoblots of Nogo-A, NgR1 and LOTUS in the olfactory bulb at E14, 15, 16, 17, 18 and P0. WT, LOTUS-KO and NgR1-KO indicate protein lysates from wild-type mice at P305, *lotus*-deficient mice at P237 and *ngr1*-deficient mice at P91, respectively. β-actin is used as an internal control protein. (**b**) The expression levels of Nogo-A, NgR1 and LOTUS are quantified by the intensity of each protein immunoblot and normalized to the intensity of β-actin. The significance level was analyzed by performing a Kruskall-Wallis test with Dunn’s multiple comparison analysis. (Nogo-A: n = 4 experiments; LOTUS: n = 3 experiments; NgR1: n = 3 experiments).

**Figure 3:**
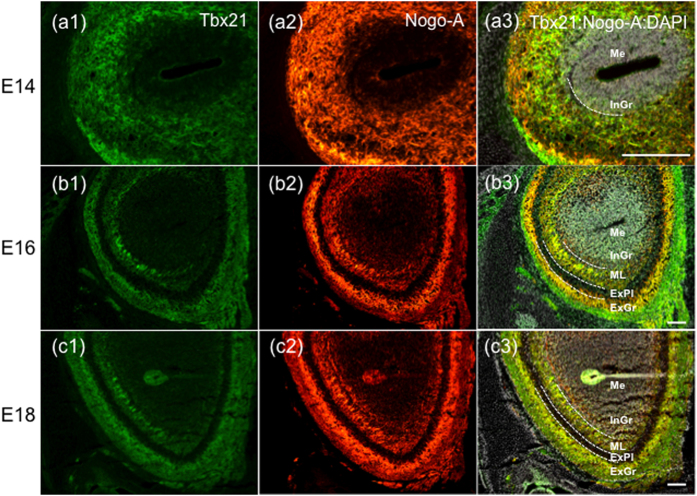
Expression of Nogo-A in mitral/tufted cell layer of the developing OB. (**a–c**) Fluorescent immunohistochemistry of Tbx21 (**a1,b1,c1**), Nogo-A (**a2,b2,c2**) and their merged image with DAPI (**a3,b3,c3**) in the developing OB of wild type mice at E14, 16, and 18. Nogo-A and Tbx21 were co-expressed outside the internal granular layer (**a3**). At E16 and E18, Nogo-A and Tbx21 were co-expressed at the mitral cell layer, the external plexiform layer and the glomerular layer (**b3,c3**). Scale Bars: 100 μm. Dotted lines are the borders between each cell layer. Me: medulla, InGr: internal granular layer, MiCe: mitral cell layer, ExPl: external plexiform layer, GlLa: glomerular layer.

Previously, we reported that LOTUS, NgR1 and Nogo-A were expressed in OB neurons and the LOT at E12 and E13^9^. In this study, we examined the expression levels of LOTUS, NgR1 and Nogo-A in the OB after E13 by immunoblotting. These three molecules were expressed after E13 in the mouse OB. The expression level of Nogo-A in the OB significantly increased during axonal collateral formation at E17 and E18 (Fig. 2). In contrast, the expression levels of LOTUS and NgR1 remained statistically unchanged, although the expression level of LOTUS showed a tendency to increase at E17 and E18 (Fig. 2). Moreover, immunohistochemistry revealed that Nogo-A was co-expressed with Tbx21 at E14, 16 and 18 (Fig. 3), which is specifically expressed in mitral and tufted cells but not in granular cells^19^, suggesting that Nogo-A was expressed in the mitral/tufted cell layer of the OB from E14 to E18. Because the LOT is formed by axons from mitral and tufted cells^20^, these results suggest that the expression of Nogo-A is developmentally increased from E17 in the LOT.

### Knockdown of Nogo-A induces a reduction in collateral branching in cultured OB neurons

We have previously reported that LOTUS antagonizes NgR1 signaling^9^,^15^. Therefore, we predicted that NgR1 function is induced by an increase of Nogo-A and that a decrease in LOTUS may be involved in axonal collateral branching of the LOT. Then, we examined whether Nogo-A and LOTUS regulate axonal branching in cultured OB neurons from E14 mice. Using immunocytochemistry, we first found that all cells expressing Nogo-A were also Tbx21- (mitral and tufted cell marker) and Tau-1-immunopositive (Supplementary Fig. S1), suggesting that NogoA is expressed in axons of mitral and tufted cells.

**Figure 4:**
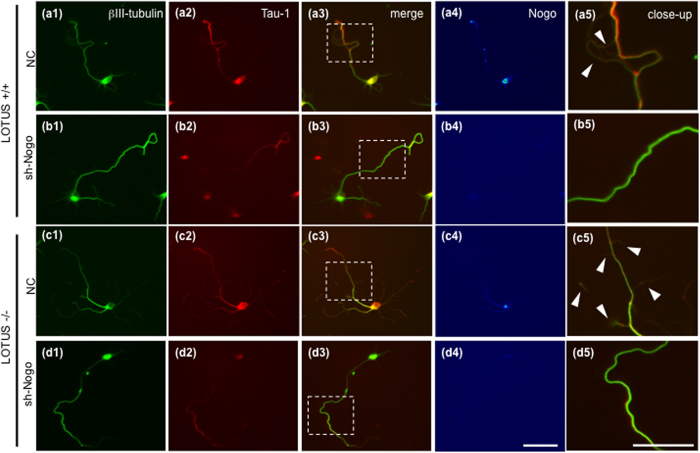
Nogo signaling mediates axonal collateral formation in cultured OB neurons. (**a1–d1**) Immunostaining of βIII-tubulin, a neuronal marker. (**a2–d2**) Immunostaining of Tau-1, an axonal marker in the same cells of (1). (**a3–d3**) Merged images of (1) and (2) and (**a4–d4**) immunostaining of Nogo-A in pseudo-color. (**a5–d5**) Dashed boxes in images of a3–d3 correspond to images of a5–d5 at higher magnification. Arrowheads indicate axonal collateral branches. Dissociated OB neurons from E14.5 wild-type mice (LOTUS+/+) (**a,b**) or *lotus*-deficient mice (LOTUS−/−) (**c,d**) were cultured for 7 days. The OB neurons were infected with lentiviral particles encoding a shRNA sequence (sh1 in b, sh3 in d) against Nogo-A mRNA (sh-Nogo) (**b,d**) or lentiviral particles encoding a negative control shRNA sequence (NC) (**a,c**). Compared with that in the LOTUS+/+ mice (NC) (**a**), axonal branching points were increased (arrowheads in c5) in LOTUS−/− mice (NC) (**c**), whereas knockdown of Nogo-A (sh-Nogo) resulted in reduction in branching points (**b,d**). Scale bars: 50 μm.

**Figure 5:**
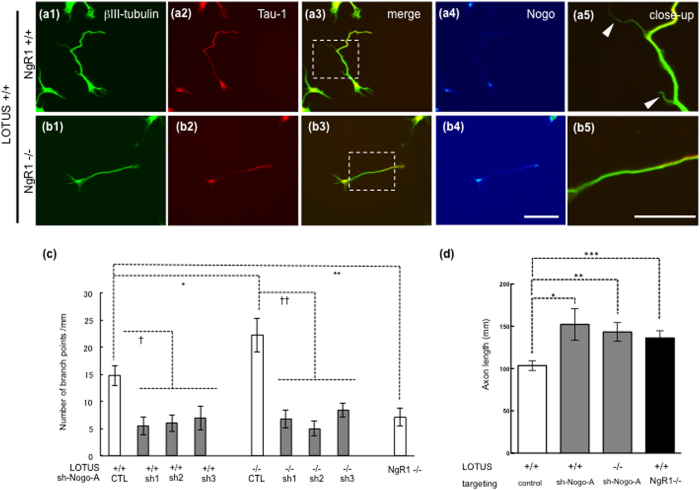
NgR1 mediates axonal collateral formation in cultured OB neurons. (**a1,b1**) Immunostaining of βIII-tubulin, a neuronal marker. (**a2,b2**) Immunostaining of Tau-1, an axonal marker in the same cells of (1), (**a3,b3**) merged images of (1) and (2), (**a4,b4**) and immunostaining of Nogo-A in pseudo-color. (**a5,b5**) Dashed boxes in images of a3 and b3 correspond to images of a5 and b5 at higher magnification, respectively. Arrowheads indicate axonal collateral branches. Dissociated OB neurons from E14.5 wild type mice (NgR1+/+) (**a**) or *ngr1*-deficient mice (NgR1−/−) (**b**) were cultured for 7days. Branching points in *ngr1*-deficient (NgR1−/−) mice were decreased in comparison with the wild type. (**c**) Quantitative analysis of axonal branching points in cultured OB neurons. Significance, indicated by (*), was obtained by performing Student’s unpaired t-test. *is *P* = 0.0437 (LOTUS+/+ versus LOTUS−/− (n = 4)), ** is *P* = 0.0029 (NgR1+/+ versus NgR1−/− (n = 3)), and comparisons indicated by (†) were obtained by performing a one-way ANOVA test with all pairwise multiple comparisons test (Tukey’s test). † is *P* < 0.05. †† is *P* < 0.000001. Scale bars: 50 μm.(**d**) Quantitative analysis of axon lengths in cultured OB neurons. Axon lengths of Tau-1-positive neurites were measured using NIH ImageJ software. Nogo-A knockdown (sh-Nogo, using sh-1 in LOTUS+/+ and sh-3 in LOTUS−/−) resulted in the increase of axon length. Similarly, axon lengths in *ngr1*-deficient (NgR1−/−) mice were increased compared to those in the wild-type mice. Significance was obtained by performing one-way ANOVA with Holm-Sidak’s multiple comparison test. *P = 0.0192 (control versus sh-Nogo treatment (n = 17–34 axons)); **P = 0.0218 (wild type versus NgR1−/− (n = 4 littermates)); ***P = 0.0229 (wild type versus LOTUS−/− (n = 5 littermates)).

We observed collateral branches of OB neurons for 7 days *in vitro* and found that the collateral branches from *lotus*-deficient (LOTUS−/−) mice were increased in comparison to that from wild-type mice (Figs 4a,c,5c), whereas the collateral branches from *ngr1*-deficient (NgR1−/−) mice were decreased in comparison to wild-type mice (Fig. 5a,b,c). These findings suggest that NgR1 function is involved in the collateral branching. Because we predicted that NgR1 function induced by an increase in Nogo-A may be involved in axonal collateral branching, we next examined whether the increase in collateral branching is induced by Nogo-A signaling. To address this issue, we examined the effect of Nogo-A knockdown on collateral branching of cultured OB neurons using lentiviral transduction particles encoding shRNA sequences that silence Nogo-A mRNA. We validated the effectiveness of three Nogo-A shRNAs (sh-Nogo-A: sh1, sh2, sh3) used in these experiments (Supplementary Fig. S2) by analyzing Nogo-A-expression intensity with immunostaining (Supplementary Fig. S3). We then regarded cells in which the Nogo-A expression level was decreased <50% as Nogo-A-knocked down cells and analyzed the number of axonal branching points in these cells. Because many granule cells in the OB lack an axon, we selected the Nogo-A-knocked down cells that were also positive for Tau-1(axonal marker) (Supplementary Fig. S1). The axonal branching points were significantly decreased by Nogo-A knockdown in the wild-type neurons (LOTUS+/+) (Figs 4b,5c) and in NgR1−/− mice (Fig. 5b,c). Furthermore, axonal branching points were similarly decreased by Nogo-A knockdown in LOTUS−/− mice (Figs 4d,5c). Lack of LOTUS (LOTUS−/−) may suppress the antagonistic action to NgR1. It was found that an increase in the axonal branching points was induced by endogenous Nogo-A through the binding to NgR1 in neurons from LOTUS−/− mice (Figs 4c,5c). Thus, Nogo signaling controlled by LOTUS may regulate axonal collateral branching in the LOT.

We also examined the effects of Nogo-A knockdown by shRNA and genetic *ngr1*-knockout (NgR1−/−) on axonal growth in cultured OB neurons. As expected, the data showed an increase in axonal growth in both Nogo-A knockdown neurons and NgR1−/− neurons (Fig. 5d). These findings suggest that Nogo signaling through the NgR1 appears to inhibit axonal growth but has a different effect on the promotion of axonal branching.

### Loss of NgR1 decreases axonal collateral branching in the developing LOT

**Figure 6:**
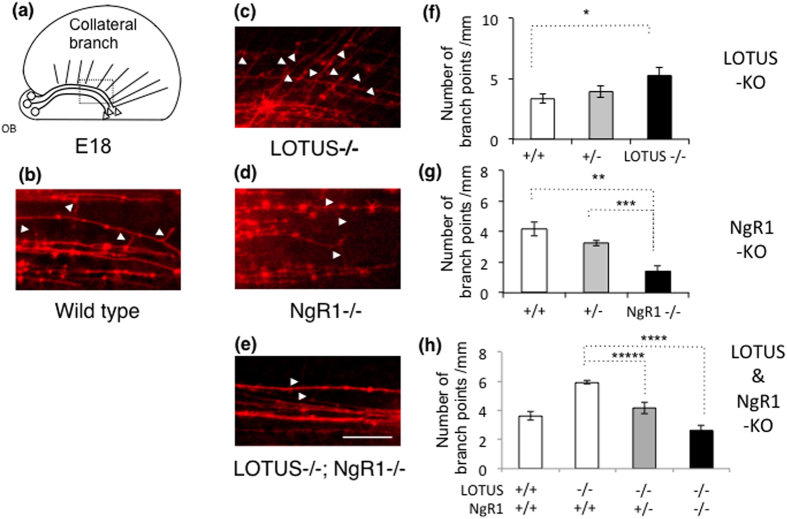
Functions of LOTUS and NgR1 in axonal branching of the LOT *in vivo*. (**a**) Schematic drawing of the lateral view of axonal branching in the LOT. The dashed square indicates the position of branch point analysis. (**b–e**) Lateral views of LOT axonal branching in whole brain from *lotus*-deficient mice (LOTUS−/−) and *ngr1*-deficient (NgR1−/−) mice at E18. Compared to wild-type mice (**b**), the axonal branching points of the LOT were increased (white arrowheads) in LOTUS−/− (**c**), whereas the branching points in NgR1−/− were decreased (**d**). Abnormally increased axonal branching observed in LOTUS−/− was rescued in double homozygous mutants of *lotus* and *ngr1* (LOTUS−/−; NgR1−/−) (**e**). Scale bar: 50 μm. (**f–h**) Quantitative analysis of branching points in the LOT. (**f**) Comparison of wild-type, LOTUS+/− and LOTUS−/− mice. (**g**) Comparison of wild-type, NgR1+/− and NgR1−/− mice. (**h**) Comparison of wild-type, NgR1+/− and NgR1−/− mice in the background of LOTUS−/− mice. Significance, indicated by (*), was analyzed using a Tukey’s one-way ANOVA test. (**f**) * is *P* = 0.031 (LOTUS+/+ versus LOTUS−/− (n = 5 littermates)). (**g**) ** is *P* = 0.00008 (NgR1+/+ versus NgR1−/− (n = 4 littermates)) and *** is *P* = 0.0016 (NgR1+/− versus NgR1−/− (n = 4 littermates)). (**h**) **** is *P* = 0.0001 (LOTUS−/−; NgR1+/+ versus LOUTS−/−; NgR1−/− (n = 3 littermates)) and ***** is *P* = 0.0076 (LOTUS−/−; NgR1+/+ versus LOTUS−/−; NgR1+/− (n = 3 littermates)).

Finally, we examined axonal collateral branching *in vivo* as visualized by DiI staining, in which DiI dye was injected into the OB at E18 when LOT axonal branching was developmentally established. In LOTUS−/− mice, collateral branches of axons in the LOT were significantly increased when compared to that in wild-type mice (Fig. 6c,f). In contrast, collateral branches of the LOT in NgR1−/− mice were significantly decreased in comparison to the wild-type mice (Fig. 6d,g). Moreover, we examined the branching points of the LOT in double mutants of LOTUS- and NgR1-knockout mice (LOTUS−/−; NgR1−/−). Axonal branching of the LOT induced by the loss of LOTUS appears to be caused by NgR1-mediated signaling because collateral branches of the LOT were reduced in double mutants of LOTUS−/−; NgR1−/− when compared to that of single mutant LOTUS−/− mice (Fig. 6e,h). These results suggest that LOTUS-mediated NgR1 function may regulate axonal branching through modulation of Nogo-NgR1 signaling.

## Discussion

In this study, we show that endogenous Nogo-A signaling through NgR1 induces axonal branching and that developmental up-regulation of Nogo-A promotes collateral formation of the LOT during the late development period. Recent studies have shown that Nogo-NgR1 signaling plays a key role in axonal branching^16^,^17^,^18^. Deng *et al*. reported that axonal branches are reduced in rat embryonic hippocampal neurons cultured on exogenous Nogo-A-coated dishes^18^. Because the methodology in this previous report is different from that used in the current study, this finding is inconsistent with our data. In contrast, it has recently been reported that endogenous Nogo-A induces axonal branching and inhibits axonal growth in cultured dorsal root ganglion neurons from rodents^16^. Similarly, our data support the hypothesis that Nogo signaling through NgR1 promotes axonal branching and inhibits axonal growth.

We examined the effect of the loss of endogenously expressed Nogo-A on axonal branching in cultured OB neurons by shRNA. When we analyzed the effect of exogenous Nogo-A using Nogo-A-coated beads on cultured OB cells, we found no effect of Nogo-A on axonal branching (data not shown). Therefore, the elimination of endogenously expressed Nogo-A may efficiently decrease axonal branches in cultured OB neurons. In another recent study, inhibition of endogenous Nogo and NgR1 functions by neutralizing antibodies against these molecules in cultured dorsal root ganglion neurons from rodents resulted in a reduction in axonal branches^16^. This previous report is consistent with our data. Moreover, axonal branches of OB axons were reduced in NgR1−/− mice both *in vitro* and *in vivo* in this study, suggesting NgR1-mediated signaling is required for axonal branching. Taken together, our data suggest that endogenous Nogo-A may induce axonal branching.

We found that loss of LOTUS (in LOTUS−/−) induced an abnormal increase in axonal branches (Fig. 6c,f), whereas simultaneous loss of NgR1 with LOTUS (in LOTUS−/−; NgR1−/−) rescued the abnormal increase in axonal branches and decreased axonal branches both *in vitro* and *in vivo* (Fig. 6e,h). These findings strongly suggest that NgR1-mediated signaling induces axonal branching. Although we have not examined *nogo-deficient* mice *in vivo* due to mouse unavailability, shRNA experiments performed *in vitro* clearly revealed that Nogo-A is required for the induction of axonal branching (Figs 4,5). We confirmed that Nogo-A, NgR1 and LOTUS were expressed in the mouse OB after LOT formation (E14), and expression of Nogo-A was up-regulated in the OB (Fig. 2), although immunoblotting experiments revealed that the expression levels of LOTUS in the OB almost remained unchanged. Therefore, axonal branching is considered to be developmentally controlled by Nogo-A-NgR1 signaling exerted by Nogo-A up-regulation and possibly associated with the overcoming of the NgR1 antagonistic action of LOTUS. Because Nogo-A showed a spotted expression in axons (Supplementary Fig.S1), it is also possible that the acute spatiotemporal increase of Nogo-A in the LOT may induce axonal collateral formation and play an important role in the development of the olfactory system. We speculate that during the early developmental period (E12-E16), unchanged expression of the three molecules investigated here mainly promotes axonal elongation of the LOT; then, during the late developmental period (E17-E18), Nogo-A up-regulation in the OB may activate Nogo-A-NgR1 signaling, thereby inducing axonal branching.

A previous study has reported that anosmin-1 encoded by KAL-1, the gene responsible for Kallman syndrome, is involved in LOT development. Loss of anosmin-1 induces a reduction in axonal branching in the embryonic rat LOT^5^. It is well known that the Rho family of molecules acts downstream of anosmin-1 signaling^21^. Therefore, Rho activation may be required for axonal branching in LOT development. In fact, previous studies have demonstrated that activation of RhoA induces axonal branching in cortical neurons^22^ and dorsal root ganglion neurons through Nogo-NgR1 signaling. Thus, Nogo-A-mediated RhoA activation induces axonal branching because Nogo-NgR1 signaling induces RhoA activation. We speculated that the signaling pathways of Nogo-A and anosmin-1 may complement each other to induce axonal branching of LOT.

Nogo-A and NgR1 have been studied extensively in CNS injury and repair. Although it has been shown that Nogo-A and NgR1 are expressed in the developing brain, little is known of their physiological functions^23^. Our present study has shown that Nogo-NgR1 signaling is physiologically important in the collateral axonal branching of LOT in the developing brain.

## Methods

### Animals

Pregnant C57BL/6 J mice were purchased from Charles River Co. (Japan, Inc.), and *lotus/crtac1b* and *ngr1* mutant mice were housed in a standard mouse facility and provided with an autoclaved diet and water. Throughout the experimental procedures, all efforts were made to minimize the number of animals used and their suffering. The experimental procedures were approved by the institutional animal care and use ethical committee of Yokohama City University and were carried out in accordance with the approved guidelines. LOTUS and NgR1 mutants were assessed in C57BL/6j background. Single mutant mice were bred with each other and intercrossed to obtain double heterozygous mice. The respective genotypes were then intercrossed among themselves to give more homozygous mutants, single mutants, and wild-type cousins for experimentation.

### Antibodies

The monoclonal hamster antibody against mouse-LOTUS (H24G11-mAb)^9^, monoclonal mouse antibody against rat-LOTUS (No.52-1E, ITM), monoclonal hamster antibody against Nogo (NG1-mAb) (kind gift from T. Hirata at the National Institute of Genetics, Mishima, Japan)^24^, and monoclonal guinea pig antibody against Tbx21 (kind gift from Y. Yoshihara at the RIKEN Brain Science Institute, Wako, Japan)^25^ were used. The monoclonal mouse antibody against Nogo-A (Millipore, Temecula, CA, USA), polyclonal goat antibody against mouse NgR1 (R&D Systems, Minneapolis, MN, USA), polyclonal rabbit antibody against βIII-tubulin (Genscript, Piscataway, NJ, USA), monoclonal mouse antibody against Tau-1 (Millipore), polyclonal rabbit antibody against Tau-1 (Santa Cruz Biotechnology, Dallas, TX, USA), monoclonal mouse antibody against β-actin (Sigma-Aldrich, St. Louis, MO, USA), biotinylated goat antibody against hamster IgG (Jackson ImmunoResearch, West Grove, PA, USA), Cy3-conjugated goat antibody against hamster IgG (Jackson ImmunoResearch), biotinylated donkey antibody against goat IgG (Jackson ImmunoResearch), Alexa488-labeled goat antibody against mouse IgG (Invitrogen, Carlsbad, CA, USA), Alexa488-labeled donkey antibody against guinea pig IgG (Jackson ImmunoResearch), Alexa594-labeled goat antibody against mouse IgG (Jackson ImmunoResearch), Alexa647-labeled goat antibody against rabbit IgG (Invitrogen), horseradish peroxidase (HRP)-conjugated donkey antibody against rabbit IgG (GE Healthcare), HRP-conjugated sheep antibody against mouse IgG (GE Healthcare, Chicago, IL, USA), HRP-conjugated donkey antibody against goat IgG (Jackson ImmunoResearch) and HRP-conjugated donkey antibody against mouse IgM (Jackson ImmunoResearch) were obtained commercially.

### Primary dissociated culture of mouse OB neurons

Dissected mouse E14 OBs were dissociated by trypsinization with 0.25% trypsin (GIBCO/BRL Life Technologies, Grand Island, New York, USA) for 5 min at 37 °C in Hank’s balanced salt solution without Ca^2+^ and Mg^2+^ (Invitrogen) and cultured on poly-L-lysine (100 μg/ml, Wako)-coated 96-well plastic culture plates (Greiner Bio-One, Kremsmuenster, Austria) with Neurobasal medium (Invitrogen) containing 1% GlutaMax (GIBCO/BRL) and 2% B27 supplement (Invitrogen) at a density of 1 × 10^4^ cells per well. The neurons were cultured at 37 °C under 5% CO_2_ for 7 days.

### Immunoblotting

For immunodetection of LOTUS, NgR1 and Nogo-A, sample lysates were prepared from the OB from E15, E16, E17 and E18 mouse embryos and P0 postnatal mice. Proteins (20 μg per lane) were subjected to SDS-polyacrylamide gel electrophoresis (8% gel) in Laemmli buffer system. After electrophoretic transfer to a polyvinylidene fluoride membrane (Millipore), proteins were blocked with 5% skim milk in Tris-buffered saline (TBS) containing 0.1% Tween-20 for 1 h at room temperature (RT) and then probed with the mouse anti-rat LOTUS antibody (ITM, 1:1000 dilution), anti-NgR1 antibody (R&D Systems, 1:1000 dilution), anti-Nogo-A antibody (Millipore, 1:1000 dilution) or anti-β-actin antibody (Sigma-Aldrich, 1:10000 dilution) for 12 h at 4 °C. Then, they were incubated with HRP-conjugated anti-mouse IgG (GE Healthcare, 1:1000 dilution for anti-rat LOTUS antibody, 1:10000 dilution for anti-β-actin antibody), HRP-conjugated anti-goat IgG (Jackson ImmunoResearch, 1:1000 dilution), HRP-conjugated anti-mouse IgM antibodies (Jackson ImmunoResearch, 1:1000 dilution) for 1 h at RT and visualized with the electrochemiluminescence kit (Takara, Kusatsu, Japan; GE Healthcare) using an imager (Image Quant 400, GE Healthcare).

### Immunostaining of mouse OB

The dissected embryonic telencephalons were fixed with 4% paraformaldehyde (PFA) (Sigma-Aldrich) in phosphate buffered saline (PBS) overnight at 4 °C and immersed in 30% sucrose in PBS for 1 day. Coronal sections at 20 μm in thickness were made with a cryostat (HM550, Thermo Fisher Scientific, Waltham, MA, USA). The sections were blocked with 5% normal horse serum (Vector Laboratories, Burlingame, CA, USA) in Tris-buffered saline (TBS) containing 0.3% Triton X-100 for 1 h and incubated with primary antibodies overnight at room temperature (RT). Primary antibodies used were as follows: guinea pig anti-Tbx21 (Yoshihara *et al*.^25^, 1:1000 dilution)^25^, mouse anti-Nogo-A (Tozaki *et al*.^24^, 1:100 dilution)^24^, and DAPI (WAKO, Osaka, Japan, 1 μg/ml). The sections were washed 3 times with TBS containing 0.3% Triton X-100, followed by incubation with fluorescence-conjugated secondary antibodies for 1 h at RT. Secondary antibodies used were Cy3-conjugated anti-hamster secondary antibodies (1:300 dilution) and Alexa488-conjugated anti-guinea pig secondary antibodies (1:300 dilution). The sections were observed by epifluorescence using a microscope (IX-73, Olympus, Tokyo, Japan).

### Immunostaining of whole-mount forebrain

Telencephalons were fixed with 4% PFA in PBS for 2 h at RT, additionally fixed for 1 h at 67 °C to inactivate endogenous alkaline phosphatase (AP), and then washed 3 times with TBS containing 5% Tween-20 (TBS-T) for 20 min at RT. The specimens were first incubated in 5% skim milk in TBS-T for 1 h at RT to block nonspecific binding of antibodies, incubated overnight at 4 °C with anti-Nrp1 (Sato *et al*.^9^, 1:10000 dilution, 20 nM)^9^ or anti-LOTUS (Sato *et al*.^9^, H24G11-mAb, 1 μg/ml)^9^ antibodies, and then washed 3 times with TBS-T for 20 min at RT. The specimens were incubated with biotinylated secondary antibodies (Jackson ImmunoResearch, 1:5000 dilution) for 2 h at RT and then washed 3 times with TBS-T for 20 min at RT, followed by incubation with the Vectastatin ABC-AP kit (Vector Labs., 1:5000 dilution) for 2 h at RT. After washing with TBS-T 3 times for 20 min at RT, immunodeposits of AP activity were detected by color reaction with NBT/BCIP. The specimens were observed using a Leica microscope (MZ-12, Leica, Wetzlar, Germany) and digital images were obtained with a CCD camera (DP70, Olympus). In all cases, matched control immunostaining was carried out with non-immune IgG or without the presence of the primary antibody.

### Immunostaining of mouse OB cultured neurons

The cultured dissociated neurons were fixed in warmed 4% PFA in culture medium for 10 min at 37 °C, followed by 10 min at RT. After rinsing with PBS, the explants were blocked with 1% bovine serum albumin (Sigma-Aldrich) in PBS containing 0.1% Triton X-100 for 1 h at RT, probed with primary antibodies against βIII-tubulin (Genscript, 0.2 μg/ml), mouse anti-Tau-1 (Millipore, 1:1000 dilution) or rabbit anti-Tau-1 (Santa Cruz Biotechnology, 1:100 dilution), Tbx21 (Yoshihara *et al*.^25^, 1:1000 dilution)^25^, and mouse anti-Nogo-A (Tozaki *et al*.^24^, NG1-mAb, 1:10 dilution)^24^ overnight at 4 °C, and then incubated with fluorescence-conjugated secondary antibodies for 1 h at RT. Cells were observed by epifluorescence using a microscope (IX-73, Olympus). Digital images were obtained with a CCD camera (DP80, Olympus) and analyzed with a personal computer with MetaMorph (Molecular Devices, Sunnyvale, CA, USA) and ImageJ (NIH) software.

### DiI tracing

To label the LOT, small crystals of DiI (D3911, Invitrogen) were implanted into the center of the OB in E18 mouse embryos fixed with 4% PFA in PBS. The telencephalons were incubated at 37 °C for approximately 3 weeks. Specimens were observed using a Leica microscope (MZ-12, Leica) and a Keyence microscope (BZ-8100, Keyence, Osaka, Japan), and digital images were obtained with a CCD camera (Olympus DP70, Olympus).

Collateral branching points in the axons were counted and divided by the total axon length. The collateral index was given as the number of branching points (countable branching points over 10 μm collateral branch) per 1 mm of an axon.

### Knockdown of mRNA

Lentiviral transduction particles encoding short hairpin RNA (shRNA) sequences that silence Nogo-A mRNA expression (GenBank accession number NM_194054.3. Clone ID sh1:TRCN0000071688, sh2:TRCN0000375427, sh3:TRCN0000379233, Sigma MISSION shRNA) (Fig. 1S) or do not silence the expression of any genes (as negative control: SHC002V, Sigma MISSION shRNA) were purchased from Sigma MISSION shRNA–RNA interference as frozen lentivirus stocks. Dissected mouse E14 OB neurons were dissociated and cultured at 37 °C for 24 h, exposed to the lentivirus at 1 IU/cell for 6 days, and then fixed and immunostained.

Collateral branching points in the axons were counted and divided by the axon length (removed axons under 100 μm in length). The collateral index was given as mentioned above.

## Additional Information

**How to cite this article**: Iketani, M. *et al*. Axonal branching in lateral olfactory tract is promoted by Nogo signaling. *Sci. Rep.* **6**, 39586; doi: 10.1038/srep39586 (2016).

**Publisher's note:** Springer Nature remains neutral with regard to jurisdictional claims in published maps and institutional affiliations.

## Supplementary Material

Supplementary Information
