# Supplementary material for: Axonal branching in lateral olfactory tract is promoted by Nogo signaling
Source: Sci Rep. 2016 Dec 21;6:39586. doi: 10.1038/srep39586 (PMC5175167; doi:10.1038/srep39586)
Supplement: Supplementary Information [file srep39586-s1.pdf]

Supplementary information

Axonal branching in lateral olfactory tract is promoted by Nogo signaling

Masumi Iketani[1, +], Takaakira Yokoyama[1, 2,+], Yuji Kurihara[1],

Stephen M. Strittmatter[3], Yoshio Goshima[4], Nobutaka

Kawahara[2] and Kohtaro Takei[1,*]

[1]Molecular Medical Bioscience Laboratory, Department of Medical Life Science, Yokohama City University Graduate School of Medical Life Science, Yokohama 230-0045, Japan,

[2]Department of Neurosurgery, Yokohama City University Graduate School of Medicine, Yokohama 236-0004, Japan,

[3]Department of Neurology and Section of Neurobiology, Yale University School of Medicine, New Haven, CT 06520, USA,

[4]Department of Molecular Pharmacology and Neurobiology, Yokohama City University Graduate School of Medicine, Yokohama 236-0004, Japan.

*Correspondence: Kohatro Takei

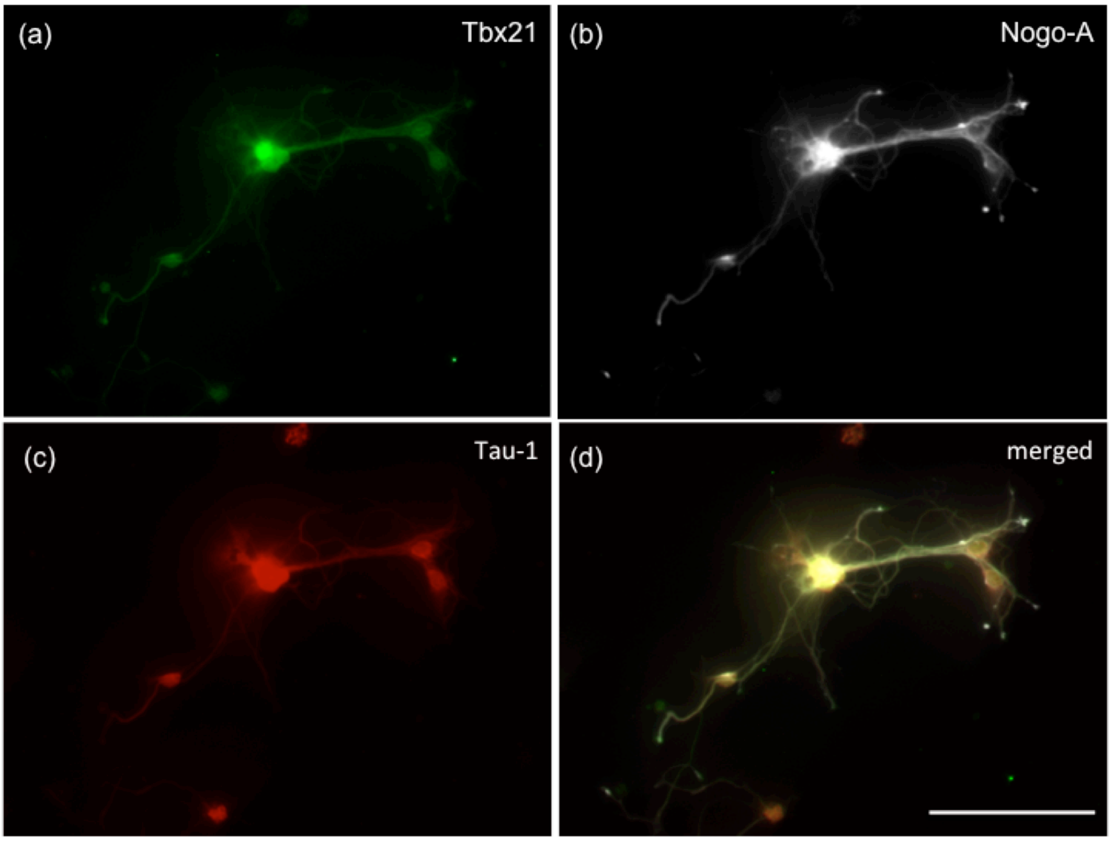

Supplementary Figure S1: Nogo-A is expressed in Tbx21- and Tau-1-immunopositive cells. Fluorescent immunocytochemistry of Tbx21 (a), Nogo-A (b) and Tau-1 (c) in OB cultured neurons. Most cells (more than 90%) expressed Nogo-A also co-expressed Tbx21 and Tau-1, showing that Nogo-A expressed cells are originated from mitral/tufted cell. Bar: 100 μm.

No.	Nucleotide number	shRNA expression sequence
		sense strand / loop / anti-sense strand
**sh1**	3565-3585	CCGGGCAGTGTTGATGTGGGTATTTCTCGAGAAATACCCACATCAACACTGCTTTTTG
**sh2**	3411-3431	CCGGCCACCCATTCAGGGCATATTTCTCGAGAAATATGCCCTGAATGGGTGGTTTTTG
**sh3**	3676-3696	CCGGCAGGCGCAGATAGATCATTATCTCGAGATAATGATCTATCTGCGCCTGTTTTTG

Supplementary Figure S2: shRNA sequences silencing Nogo-A mRNA expression. The sequences are expressed by lentiviral particles to silence the expression of mouse Nogo-A mRNA (NM_194054.3). The numbers described on the left side of each sequence show the nucleotides number of NogoA mRNA (NM_194054.3) that the shRNA targets. They are cloned in shRNA lentivirus vectors and lentiviral particles are provided by Sigma (Clone ID sh1:TRCN0000071688, sh2:TRCN0000375427, sh3: TRCN0000379233, Sigma MISSION shRNA).

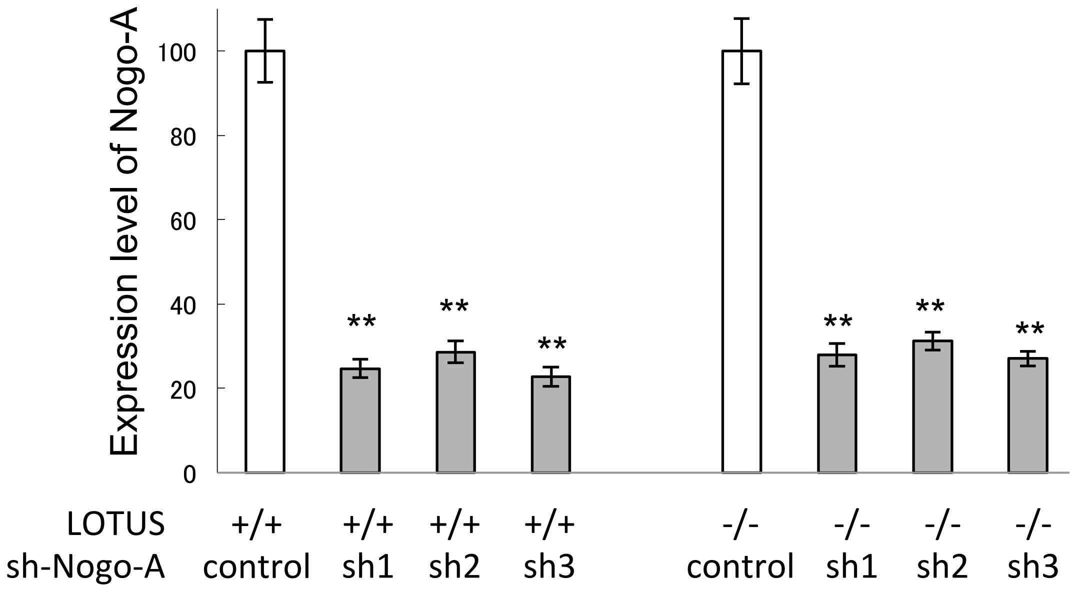

Supplementary Figure. S3: Quantitative analysis of Nogo-A expression. The expression level of Nogo-A in cultured OB neurons was visualized with immunostaining, quantified by measuring intracellular luminous intensity with the Metamorph software (Molecular Devices) and normalized to the wild-type or *lotus*-knockout neurons exposed to negative-control shRNA-expressed virus respectively. Significance was performed by Tukey one-way ANOVA test. $^{**}P < 0.01$ by Tukey one way-ANOVA test (NC, negative control versus sh-Nogo-A: 1, 2, 3).
